# Single-Cell-Derived Malignant Epithelial Programs Define Prognostic Risk and Therapeutic Vulnerability in Ovarian Cancer

**DOI:** 10.7150/jca.135548

**Published:** 2026-07-03

**Authors:** Jingwen Si, Hanbo Li, Han Zhang, Yan Shen

**Affiliations:** 1 Department of Pathology, Tianjin Central Hospital of Gynecology Obstetrics, Tianjin, China.; 2 Tianjin Chest Hospital, Tianjin University, Tianjin, China.

**Keywords:** OV, scRNA-seq, Epithelial cells, inferCNV, immunotherapy, PSMB1.

## Abstract

**Background:**

Ovarian cancer (OV) is characterized by pronounced intratumoral heterogeneity and unfavorable clinical outcomes, yet clinically applicable biomarkers for risk stratification remain limited. Although bulk transcriptomic models have been widely proposed, they often overlook the cellular origins of prognostic signals. Advances in single-cell RNA sequencing (scRNA-seq) provide an opportunity to resolve malignant epithelial diversity and identify biologically meaningful predictors.

**Methods:**

Single-cell transcriptomic datasets were integrated to construct a comprehensive epithelial landscape of OV. Copy number variation profiles inferred by inferCNV were used to distinguish malignant epithelial cells, followed by subpopulation identification and trajectory inference using Slingshot and Monocle3. Subcluster-specific gene signatures were projected onto bulk RNA-seq cohorts, and prognostic genes were screened using Cox regression and LASSO modeling to establish a multigene risk score. The model was validated across multiple independent cohorts. Multi-omics analyses, including pathway enrichment, mutational profiling, tumor mutation burden, immune features, and drug sensitivity prediction, were performed to explore biological and clinical relevance. Functional assays were conducted to validate the role of the key gene PSMB1.

**Results:**

Malignant epithelial cells exhibited substantial transcriptional heterogeneity and distinct evolutionary trajectories, revealing subpopulations associated with differential clinical outcomes. A nine-gene risk model derived from these subpopulations demonstrated robust and consistent prognostic performance across multiple datasets. The risk score was closely associated with tumor progression pathways, immune suppression, genomic instability, and reduced therapeutic sensitivity. Notably, PSMB1 was identified as a critical oncogenic factor, and its knockdown significantly inhibited proliferation and colony formation in OV cells.

**Conclusions:**

This study establishes a single-cell-informed prognostic framework that links malignant epithelial heterogeneity to clinical outcomes and therapeutic response in OV. The proposed model provides biologically interpretable risk stratification and highlights PSMB1 as a potential therapeutic target, offering new insights for precision oncology.

## 1. Introduction

OV is a highly fatal malignancy of the female reproductive system, noted for its significant heterogeneity and subtle early symptoms 1. As a result, the majority of patients are diagnosed at advanced stages 2. Despite advancements in surgery, chemotherapy, targeted therapy, and immunotherapy, the overall prognosis remains poor, with a low five-year survival rate 3. Particularly under homogenized treatment strategies, the need for precise risk stratification and individualized therapeutic guidance has become a pressing clinical challenge 4, 5.

Intratumoral heterogeneity in OV encompasses molecular and cellular variations, not just histological differences. Traditional bulk RNA sequencing technologies, which capture averaged signals from mixed populations, are limited in their ability to resolve the specific roles of distinct cellular subsets during tumor initiation and progression. scRNA-seq has recently transformed cancer research by allowing detailed analysis of intratumoral cellular heterogeneity with exceptional resolution 6. Unlike bulk transcriptomic approaches, which obscure the contributions of individual cell types, scRNA-seq allows researchers to distinguish and characterize distinct cellular populations within the tumor microenvironment 7, 8. In OV, where epithelial cells constitute a major component of the tumor mass, this technology has unveiled remarkable transcriptional diversity among epithelial subpopulations. Emerging evidence indicates that these subsets differ not only in gene expression patterns, but also in functional attributes such as proliferative potential, differentiation status, metabolic activity, and immune modulatory capacity 9, 10. Notably, some epithelial clusters exhibit stem-like phenotypes, enhanced survival capabilities, or resistance to cytotoxic insults, suggesting their potential role as key drivers of tumor progression, metastasis, and therapeutic failure. Integrating single-cell data into prognostic models and therapeutic decisions is essential to accurately reflect the biological complexity of OV. Recent single-cell and multi-omics studies have further shown that malignant tumor-cell subpopulations can reflect distinct biological programs and prognostic features, supporting the value of linking cellular heterogeneity to patient-level risk stratification 11.

While several prognostic models for OV have been proposed based on bulk transcriptomic data, most lack insights into the biological origins of their constituent genes, failing to account for the heterogeneity at the cellular level 7, 12, 13. This limits both their robustness and interpretability. This study seeks to combine scRNA-seq and bulk RNA-seq data to pinpoint crucial genes from malignant epithelial subpopulations, and develop a prognostic scoring system informed by biological data using machine learning methods. Furthermore, we systematically evaluate the association of this signature with immune contexture, mutational landscape, pathway activity, and immunotherapy responsiveness across multiple omics layers, thereby enhancing its clinical applicability and translational potential.

We developed a detailed epithelial atlas of OV by combining single-cell and bulk transcriptomic data, revealing subpopulations with different levels of malignancy. Subcluster-specific marker genes were subsequently used to derive a prognostic model with clear cellular origins through machine learning algorithms, and its predictive performance was validated across multiple independent cohorts. Multi-omics analyses further revealed that the model signature was closely associated with signaling pathway activity, tumor immune microenvironment, and therapeutic response. In addition, functional assays confirmed that the core model gene, PSMB1, plays a tumor-promoting role, offering mechanistic insights and a potential target for therapeutic intervention.

## 2. Methods

### 2.1 Summary of Public Databases Employed in the Study

The single-cell transcriptomic datasets analyzed in this study were curated from the GEO database, specifically datasets GSE154600 and GSE184880. GSE154600 includes five primary OV samples, while GSE184880 provides single-cell RNA profiles for five normal ovarian tissues and seven OV tumor specimens, enabling comparisons between malignant and non-malignant states. Bulk RNA sequencing data were obtained from both TCGA and GEO repositories. The TCGA-OV cohort was employed as the primary training set for model construction. Three independent datasets—GSE13876, GSE140082, and GSE26712—were selected from GEO to serve as external validation sets, enabling robust assessment of model performance across platforms. Before model validation and signature projection, bulk expression matrices from different cohorts were log2-transformed when necessary, matched by gene symbols, and further corrected for cross-dataset batch effects using the ComBat function in the sva R package, with cohort or platform information used as the batch variable. Clinical metadata and somatic mutation annotations (MAF files) for TCGA-OV were obtained from the Genomic Data Commons (GDC) portal using the TCGAbiolinks R package 14. To examine the model's cross-cancer applicability, pan-cancer expression profiles and corresponding clinical annotations were downloaded from the UCSC Xena platform. TIDE scores, used to predict immunotherapy responses, were obtained from the official TIDE web interface (http://tide.dfci.harvard.edu/).

### 2.2 Single-Cell Processing Pipeline and Cross-Sample Harmonization

scRNA-seq data were processed following a standard pipeline using the Seurat framework 15, 16. Prior to downstream analyses, rigorous quality control was applied based on the number of transcripts, the number of expressed genes, and the proportion of mitochondrial transcripts per cell. Cells that did not meet the defined thresholds were removed; filtering criteria are provided in Supplementary Table 1. After quality filtering, gene expression values were normalized, and genes with high variability across cells were retained for further analysis. The dataset was then scaled, and potential confounders such as mitochondrial gene expression were accounted for during preprocessing. To mitigate technical variability across samples, the Harmony algorithm was employed for batch effect correction. This integration approach allowed cells from different datasets to align within a shared low-dimensional representation. Clustering was performed in the principal component space, and cellular heterogeneity was visualized using a two-dimensional embedding. Cell identities were annotated based on the expression of canonical lineage markers, supplemented by manual verification using reference databases and published literature. Visualization of cell clusters and gene expression patterns was enhanced using the SCP package 17, ensuring high-resolution graphical outputs.

### 2.3 Mapping Intercellular Communication Networks between Normal and Tumor Tissues

To uncover intercellular communication differences between OV tissues and normal ovarian samples, we constructed signaling interaction networks using single-cell transcriptomic data. Cell-cell communication was deduced using known ligand-receptor interactions, with interaction strengths calculated separately for tumor and normal conditions. Enrichment analysis was conducted to identify signaling pathways exhibiting differential activity between the two tissue types. Subsequent analyses focused on epithelial cells, investigating their role as signaling initiators. We analyzed pathways that showed significant upregulation or downregulation in tumor-derived epithelial populations relative to normal counterparts. The analysis leveraged a high-confidence interaction database and accounted for both expression levels and interaction likelihood. Final outputs were summarized at the signaling pathway level, highlighting key communication patterns reshaped by the tumor microenvironment.

### 2.4 Genomic Instability Estimation via inferCNV in Epithelial Populations

We utilized the inferCNV package 18 in R to analyze large-scale copy number variations (CNVs) from single-cell transcriptomic data, aiming to study genomic instability in epithelial cells. Epithelial cells derived from normal ovarian tissues were designated as the reference group. By comparing gene expression intensities along chromosomal positions, relative CNV patterns were generated to reflect potential genomic gains or losses. A dual-criteria approach was employed to identify malignant epithelial cells. Cells were deemed malignant if their average CNV value exceeded 0.2 or their Pearson correlation coefficient was greater than 0.2 compared to the top 5% of cells with the highest CNV signals. This approach enhances the robustness of malignant cell detection under conditions of intratumoral heterogeneity and provides insight into chromosomal alterations characteristic of tumorigenic epithelial subsets.

### 2.5 Pseudotime Trajectory Analysis Using Slingshot and Monocle3

To elucidate the potential dynamic transitions of malignant epithelial cells during tumor progression, we performed pseudotime trajectory reconstruction using two complementary algorithms: Slingshot 19 and Monocle3 20. Slingshot was first employed to infer lineage trajectories based on predefined clustering and low-dimensional embeddings. This approach fits continuous lineage structures across clusters and assigns pseudotime values to individual cells according to their spatial progression along the inferred paths. To validate the robustness of the trajectory and explore cellular transitions within a different computational framework, Monocle3 was applied to the same set of malignant cells. This method constructs a principal graph that models the developmental landscape and orders cells along trajectories by learning the global topology of transcriptional states. Both tools enabled the reconstruction of developmental hierarchies and provided a foundation for downstream branch-specific enrichment analysis.

### 2.6 Association Analysis Between Malignant Cell Subpopulations and Patient Prognosis

To investigate the potential impact of distinct malignant epithelial subpopulations on overall prognosis, we first identified cluster-specific marker genes based on the tumor cells previously inferred by inferCNV. Representative gene sets for each malignant cluster were identified through differential expression analysis. The gene signatures were then applied to the bulk RNA-seq data from the TCGA-OV cohort. For each patient, enrichment scores corresponding to each cluster-specific gene set were calculated using a gene set scoring algorithm. Patients were categorized into high- and low-score groups according to the median enrichment score. Kaplan-Meier survival curves and log-rank tests were used to assess the prognostic value of each malignant subpopulation. This method facilitated the identification of clusters linked to adverse outcomes, offering potential insights for discovering therapeutic targets.

### 2.7 Translating Single-Cell Insights into a Multigene Risk Score

We examined the prognostic significance of specific malignant epithelial subpopulations in OV, concentrating on clusters 0, 1, and 3, previously linked to patient survival. Marker genes for each cluster were identified using Seurat's FindAllMarkers function, applying thresholds of |log2FC| > 0.25, adjusted p-value < 0.05, and min.pct > 0.25. These marker genes were then mapped to the bulk RNA-seq expression data from the TCGA-OV cohort. A univariate Cox regression analysis was conducted to identify genes significantly associated with patient survival (p < 0.05). To construct a robust prognostic model, we applied the Least Absolute Shrinkage and Selection Operator (LASSO) regression using the glmnet R package. The optimal penalty parameter lambda was determined through tenfold cross-validation. Genes selected by LASSO were further refined using stepwise regression with the Akaike Information Criterion (stepAIC) to develop the final multigene Cox regression model.

The risk score for each patient was then calculated using the following formula:

Risk score = Σ (Coefᵢ × Expᵢ)

where Coefᵢ is the regression coefficient and Expᵢ is the expression level of gene i.

Patients were categorized into high- and low-risk groups according to the median risk score. Kaplan-Meier survival analysis and log-rank tests were then performed to evaluate the prognostic performance of the model.

### 2.8 Gene Set Enrichment Analysis Highlights Molecular Distinctions Between Risk Groups

We conducted Gene Set Variation Analysis (GSVA) on all samples to evaluate hallmark pathway enrichment levels 21, utilizing gene sets from the MSigDB H collection to investigate functional differences between distinct risk groups. Patients were categorized into high- and low-risk groups according to the median risk score. Genes with differential expression were identified using criteria of |log2 fold change| > 1 and FDR < 0.05. GO Biological Process and KEGG pathway enrichment analyses were performed to determine functional patterns specific to each group. Heatmaps and bubble plots were utilized to visualize the enriched biological functions and signaling pathways.

### 2.9 Integrated Evaluation of Risk Model and Tumor Mutation Burden

We conducted a detailed analysis of the mutational landscape using the TCGA-OV cohort to explore the relationship between risk scores and somatic mutation features. MAF files were processed and visualized using the Maftools R package 22 to analyze the global mutation profile, covering mutation frequency, variant classifications, types, and SNV classes. Subsequently, we extracted mutation data for the prognostic model-associated genes across all patients and illustrated their distribution using waterfall plots to assess the mutational frequency and potential biological relevance of each gene. Patients were categorized into high- and low-risk groups based on the median risk score, and their mutational landscapes were separately plotted to compare mutational spectra. TMB, defined as nonsynonymous mutations per megabase, was compared across risk subgroups. A correlation analysis was performed to assess the relationship between TMB and the risk score. Patients were stratified by TMB and risk score, and Kaplan-Meier survival curves with log-rank tests evaluated the prognostic impact of their combined effect. This multi-layered analysis provides additional support for the interpretability and clinical relevance of the proposed prognostic model.

### 2.10 Pan-Cancer Landscape of Risk Score Correlation with Tumor Phenotypic Programs

We conducted a pan-cancer analysis using TCGA transcriptomic data from 33 solid tumors, including OV, to thoroughly assess the relationship between the constructed risk score and tumor phenotype-related pathways across different cancer types. The risk score for each sample was calculated as previously described. We utilized hallmark gene sets from the MSigDB database to quantify key tumor biological processes, specifically focusing on angiogenesis and epithelial-mesenchymal transition (EMT). GSVA was used to calculate pathway enrichment scores for each sample. Pearson correlation analysis was conducted to evaluate the associations between risk scores and pathway activities across various tumor types. The results were visualized using scatter plots, with Pearson coefficients and p-values annotated to highlight tumor-type-specific associations.

### 2.11 Cell Lines and Cell Culture

The human OV cell lines SKOV3 and ES-2 were obtained from the Cell Bank of the Chinese Academy of Sciences (Shanghai, China). All cell lines were authenticated by short tandem repeat (STR) profiling and confirmed to be free of mycoplasma contamination prior to experimentation. SKOV3 and ES-2 cells were cultured in RPMI-1640 medium (Gibco, USA) supplemented with 10% fetal bovine serum (FBS) (Gibco, USA), 100 U/mL penicillin, and 100 µg/mL streptomycin, and maintained in a humidified incubator at 37 ºC with 5% CO₂. Cells were passaged at 70-80% confluence using 0.25% trypsin-EDTA and were used within 10 passages for all experiments to ensure stability.

### 2.12 Cell Proliferation Assay (CCK-8)

To assess the impact of PSMB1 on OV cell proliferation, the Cell Counting Kit-8 (CCK-8, Beyotime, China) assay was conducted. SKOV3 and ES-2 cells transfected with siRNA were seeded into 96-well plates at a density of 5 × 10^3^ cells per well, with three technical replicates per group. From Day 1 to Day 7 post-transfection, 10 µL of CCK-8 reagent was added daily to each well, followed by a 2-hour incubation. Absorbance at 450 nm was measured using a microplate reader. Each experiment was performed in triplicate, and the mean OD values were used to generate cell growth curves over the 7-day period.

### 2.13 Colony Formation Assay

To further investigate the effect of PSMB1 on the proliferative and survival capacities of OV cells, a colony formation assay was performed. SKOV3 and ES-2 cells transfected with siRNA were seeded into 6-well plates at a density of 500 cells per well. Both control and siRNA groups were included, with three replicates each. Cells were cultured for approximately 10-14 days until visible colonies formed. The plates were gently washed with PBS, fixed in 4% paraformaldehyde for 15 minutes, and stained with 0.1% crystal violet for 30 minutes. After removing excess dye, images were captured, and colonies larger than 50 μm were counted using ImageJ software. Colony formation rates were calculated and statistically analyzed.

## 3. Results

### 3.1 Single-cell Transcriptomic Profiling of OV Samples, Including Data Preprocessing, Batch Effect Mitigation, and Cell Type Classification

**Figure 1:**
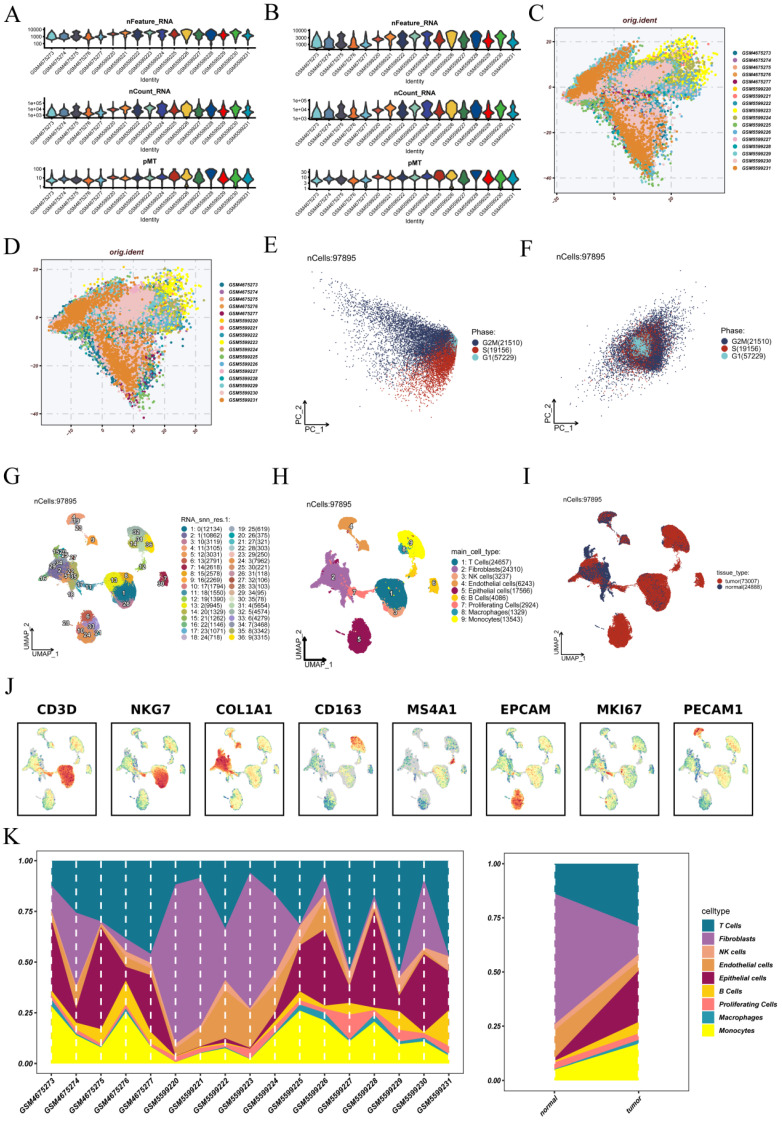
** Single-cell RNA sequencing data undergoes quality control, batch correction, and cell-type annotation.** (A-B) Distribution of gene counts (nFeature_RNA), transcript counts (nCount_RNA), and mitochondrial gene percentages (percent.mt) across all samples before and after quality control. (C-D) Visualization of sample distribution in low-dimensional space before and after batch correction using the Harmony algorithm. (E-F) Principal component analysis showing cell cycle phase distributions before and after regressing out cell cycle-related gene effects. (G) UMAP projection illustrating the spatial arrangement of transcriptionally distinct cell clusters. (H) Cell-type annotation based on canonical marker genes and reference databases, with each color representing a distinct cell lineage. (I) UMAP visualization of cells colored by tissue origin (tumor vs. normal). (J) Expression patterns of representative marker genes (e.g., CD3D, NKG7, EPCAM, MKI67) across identified cellular subpopulations. (K) Proportional distribution of major cell types across different patients and tissue sources.

We initiated our analysis by performing stringent quality control on the scRNA-seq data derived from OV samples. As illustrated in Figure 1A, the unfiltered dataset included cells with low gene counts, abnormal transcript levels, or high mitochondrial content. Applying filtering criteria centralized and standardized the distribution of nFeature_RNA, nCount_RNA, and percent.mt across samples (Figure 1B), improving data reliability for subsequent analyses. We utilized the Harmony algorithm to correct potential batch effects among samples. Prior to adjustment, cells from different samples formed distinct clusters in the low-dimensional space (Figure 1C). After integration, cells were well mixed across batches, demonstrating successful elimination of technical variation (Figure 1D). Given the confounding influence of cell cycle genes on dimensionality reduction, we regressed out these effects using Seurat's normalization pipeline. Before correction, cells at different phases exhibited biased distributions along principal components (Figure 1E). Post-regression, the impact of cell cycle was substantially mitigated, yielding a more uniform representation (Figure 1F).

Following preprocessing and integration, a total of 97,875 high-quality cells were retained for further analysis. Unsupervised clustering and UMAP projection revealed 36 distinct cellular subpopulations (Figure 1G), highlighting the extensive heterogeneity within the tumor ecosystem. Using canonical markers and reference-based classification, we identified these clusters as major cell lineages: T cells, B cells, epithelial cells, macrophages, dendritic cells, fibroblasts, and endothelial cells (Figure 1H). The spatial mapping of tissue origin indicated that the majority of cells were derived from tumor tissues rather than adjacent normal regions (Figure 1I), suggesting that the tumor microenvironment dominates the cellular composition landscape. We analyzed the expression profiles of key marker genes, such as CD3D, NKG7, EPCAM, and MKI67, which exhibited unique distribution patterns among the identified cell types (Figure 1J), supporting the accuracy of the classification. Finally, we measured the abundance of each cell type across different patients and tissue sources (Figure 1K). T cells exhibited the highest overall abundance and showed marked variability among patients. Tumor tissues were enriched with T cells, B cells, and epithelial cells, while fibroblasts and endothelial cells were comparatively sparse. These compositional shifts underscore the complex and heterogeneous immune landscape of OV.

### 3.2 Cellular Communication Heterogeneity Reveals Immune Microenvironment Reprogramming in Ovarian Cance

**Figure 2:**
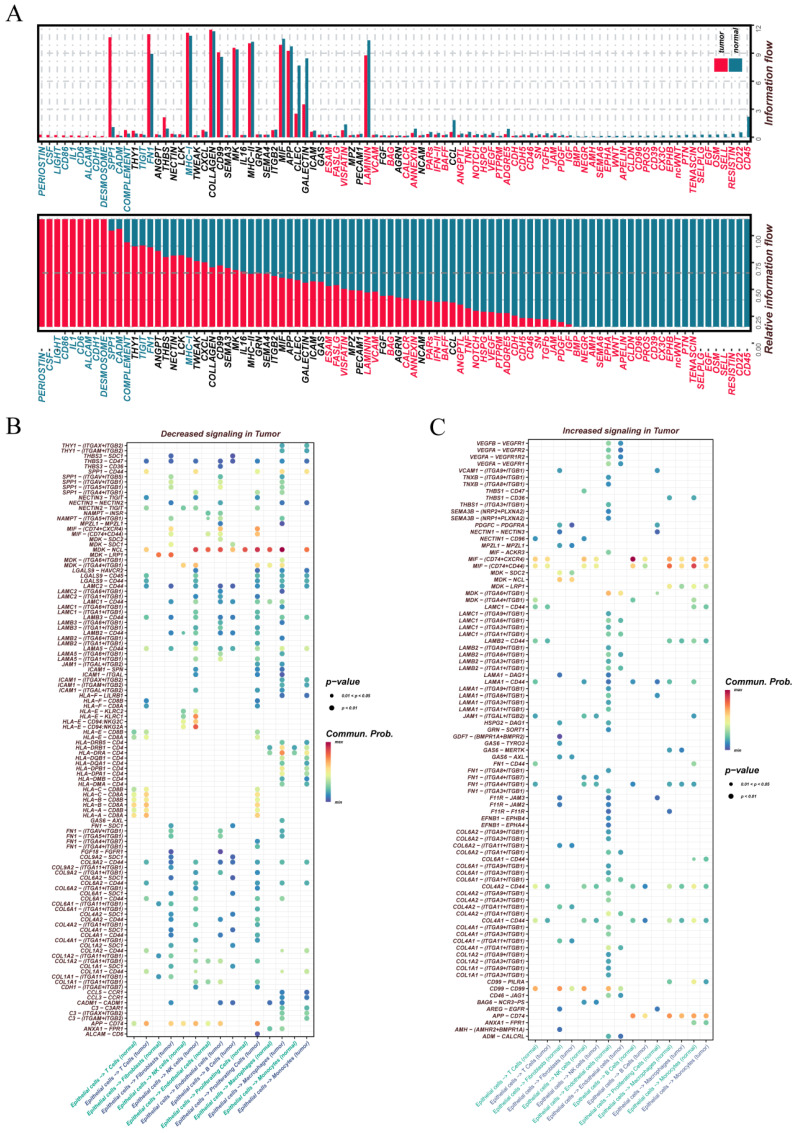
** A comparative study of cell communication patterns in tumor versus normal tissues.** (A) Bar plots illustrate signaling pathways showing significant differences in interaction patterns between tumor and normal samples. The upper panel presents the total number of detected interactions (absolute counts) for each pathway across the two tissue types. The lower panel displays the relative dominance of each pathway within either tumor or normal tissues, indicating its predominant activation context. (B) Communication networks initiated by epithelial cells in tumor tissues, highlighting pathways with increased outgoing signaling activity and their corresponding target cell populations. (C) Signaling pathways originating from epithelial cells that exhibit reduced communication strength in tumor samples, along with their respective downstream interacting cell types.

We systematically compared signaling pathway activity between ovarian tumor and normal tissues to identify differences in cell-cell communication (Figure 2A). The tumor samples exhibited enhanced signaling via SPP1, FN1, MHC-I, TIGIT, and COMPLEMENT pathways, which are associated with immunosuppression, extracellular matrix remodeling, and antigen presentation. In contrast, CD45, LAMININ, VCAM, and VEGF pathways were more prominent in normal tissues, indicating their involvement in immune surveillance and basement membrane maintenance.

Focusing on epithelial cells as signal senders, we observed that multiple outgoing interactions were markedly elevated in tumor tissues (Figure 2B). The interactions MDK-NCL, APP-CD74, MIF-(CD74+CD44), MIF-(CD74+CXCR4), and HLA-E-CD94-NKG2C are mainly associated with immune regulation and antigen recognition. Conversely, pathways related to cell adhesion—such as COL4A1-CD44, COL4A2-CD44, and CD99-CD99—were notably downregulated in tumors (Figure 2C), suggesting impaired epithelial adhesion and a potential shift toward enhanced migratory or invasive behavior.

### 3.3 Dissecting Epithelial Cell Heterogeneity Through CNV-Based Malignancy Profiling

**Figure 3:**
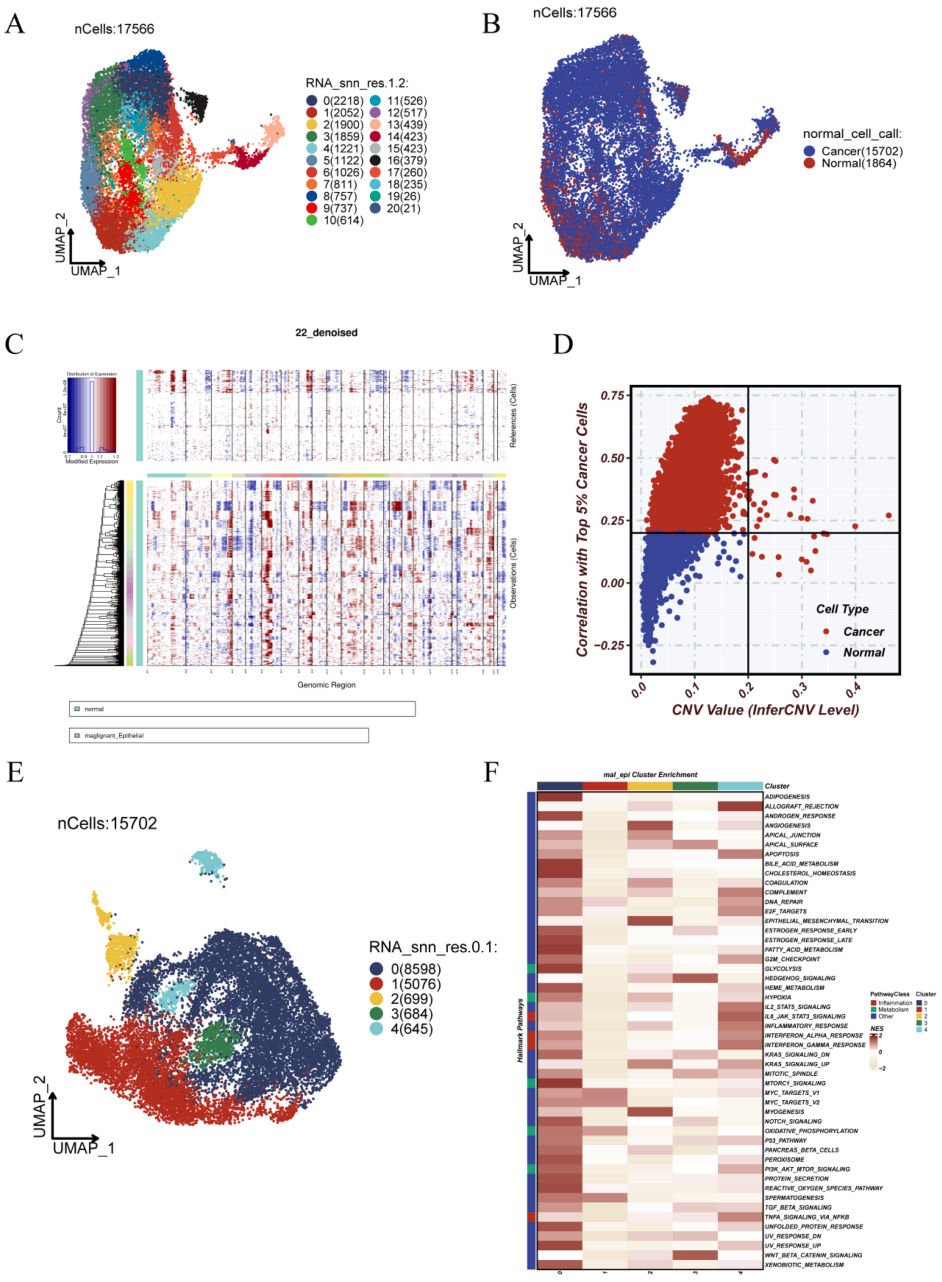
** Dissecting Epithelial Cell Heterogeneity Through CNV-Based Malignancy Profiling.** (A) UMAP-based reclustering of epithelial cells identified 20 heterogeneous subpopulations. (B)Epithelial cells were classified by tissue origin, with tumor-derived cells marked in red and normal tissue cells in blue. (C)Single-cell copy number variations (CNVs) were deduced using inferCNV. The heatmap displays genomic CNV patterns, with the upper panel representing reference (normal) cells and the lower panel showing predicted tumor cells. (D)Malignant epithelial cells were detected by analyzing CNV values and their association with the top 5% of cells exhibiting high CNV.Cells with CNV values > 0.2 or correlation > 0.2 were classified as malignant (red). (E) Reclustering of malignant epithelial cells revealed distinct substructures and intratumoral heterogeneity. (F) Heatmap of pathway enrichment across malignant subclusters, highlighting their functional divergence.

To further investigate the heterogeneity and malignant characteristics of epithelial cells, we performed reclustering analysis on the epithelial population. UMAP-based dimensionality reduction revealed 20 transcriptionally distinct subclusters, indicating substantial intragroup heterogeneity (Figure 3A). Tissue origin annotation showed that the majority of epithelial cells were derived from tumor samples, with a higher abundance compared to those from normal tissues (Figure 3B). We utilized normal tissue-derived epithelial cells as a reference to apply inferCNV for inferring large-scale chromosomal alterations at single-cell resolution. The results revealed widespread copy number gains and losses in tumor-derived cells, suggesting the presence of genomic instability (Figure 3C). Cells exhibiting either a mean CNV value greater than 0.2 or a Pearson correlation exceeding 0.2 with the top 5% high-CNV cells were classified as malignant epithelial cells (Figure 3D). Further reclustering of these malignant cells revealed additional transcriptional diversity within the tumor epithelial compartment (Figure 3E). Functional enrichment analysis demonstrated that specific subpopulations exhibited distinct pathway preferences. In particular, subcluster 0 was strongly associated with metabolic and stress-related pathways, including GLYCOLYSIS, MTORC1_SIGNALING, and REACTIVE_OXYGEN_SPECIES_PATHWAY, suggesting a role in maintaining tumor viability and potential therapy resistance (Figure 3F).

### 3.4 Developmental Trajectories Reveal Divergent Evolution of Malignant Epithelial Subpopulations

**Figure 4:**
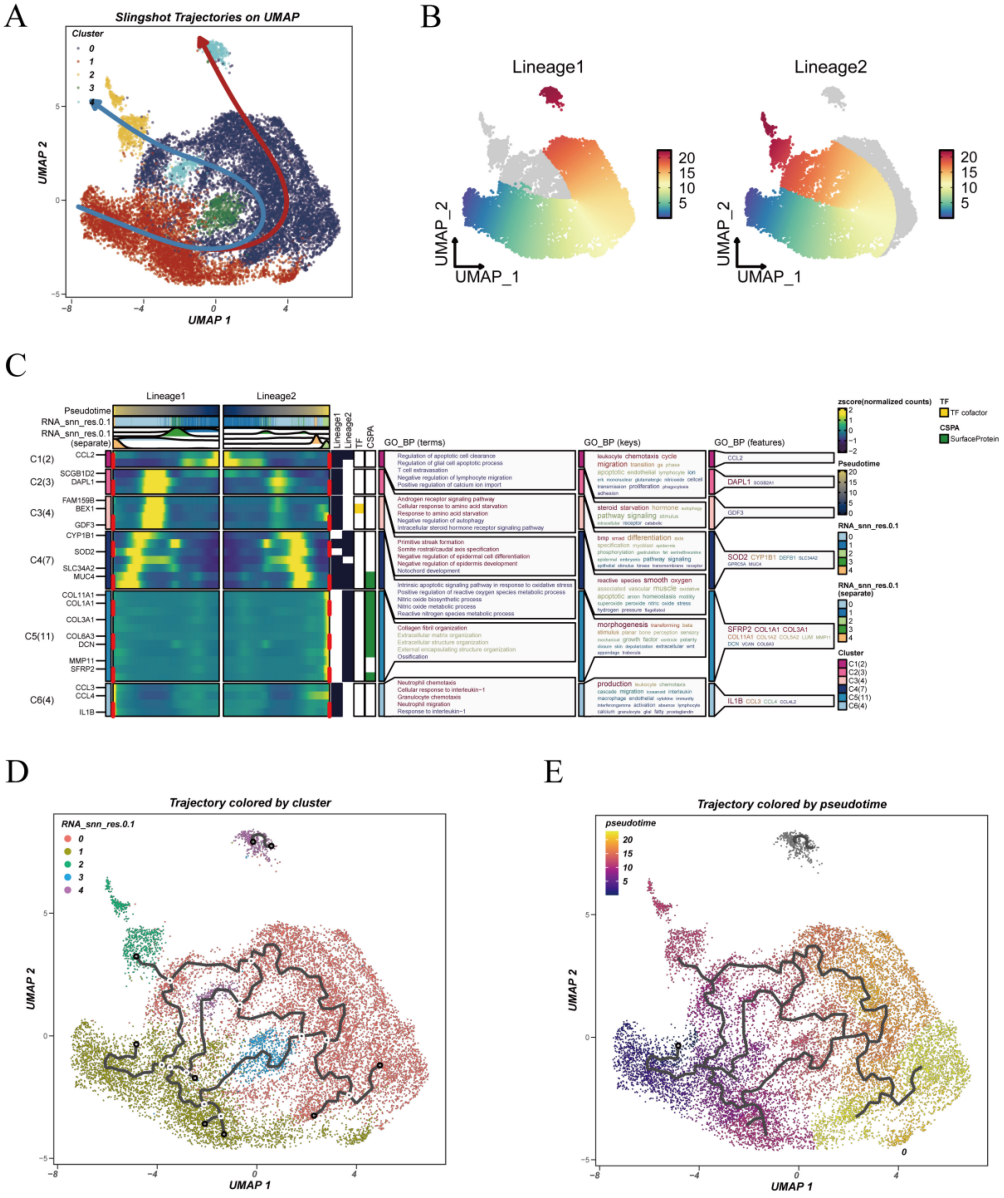
** Pseudotime trajectory analysis reveals differentiation paths of malignant epithelial cells.** (A) UMAP projection analyzed using the Slingshot algorithm reveals two primary developmental trajectories: Lineage 1 and Lineage 2. (B) Pseudotime progression of cells along each trajectory, with color gradients representing transitions from early to late pseudotime states. (C) GO enrichment analysis of subclusters along the two lineages, highlighting functional heterogeneity across biological processes, molecular functions, and cellular components. (D) Pseudotime trajectory constructed using Monocle3, with cells colored by their original clustering identities, illustrating the correspondence between trajectory and cellular states. (E) Monocle3-derived pseudotime projection colored by pseudotime values, demonstrating the directionality and continuity of the differentiation process.

To delineate the developmental trajectories of malignant epithelial cells, we employed the Slingshot algorithm for pseudotime inference. As shown in Figure 4A, two distinct lineages were identified, with Cluster 1 serving as the common root state, while Clusters 0 and 2 appeared at the terminal ends of the respective trajectories, suggesting divergent differentiation fates. This branching pattern implies that malignant cells may originate from a shared progenitor-like state and evolve along separate paths under tumor microenvironmental pressures. Figure 4B presents the pseudotime distribution of cells across the inferred trajectories, highlighting a gradual and continuous transition in cellular states.

Functional enrichment analysis comparing the two lineages is shown in Figure 4C. Genes enriched along Lineage 1 were primarily associated with the androgen receptor signaling pathway, cellular response to amino acid starvation, negative regulation of autophagy, and intracellular steroid hormone receptor signaling, suggesting an adaptive response to hormonal and nutrient signals. In contrast, Lineage 2 showed enrichment in pathways associated with cell migration, immune modulation, and extracellular matrix remodeling, suggestive of a more aggressive and potentially invasive phenotype.

To validate the robustness of the inferred trajectories, we further applied Monocle3 for pseudotime reconstruction. As shown in Figure 4D, cells were colored by their original clusters, while Figure 4E illustrates a continuous trajectory along the pseudotime axis, confirming the developmental transitions observed with Slingshot.

### 3.5 Association Analysis Between Malignant Cell Subpopulations and Patient Prognosis

We calculated enrichment scores for each patient in the TCGA-OV cohort using marker genes specific to each malignant epithelial subcluster, as illustrated in Supplement Figure 1. Patients were divided into high- and low-score groups based on the median score, followed by Kaplan-Meier survival analysis. The findings indicated a significant association between specific subclusters and patient prognosis. High enrichment in cluster 0 (p = 0.012) and cluster 2 (p = 0.082) correlated with poorer survival, while clusters 1 (p = 0.02), 3 (p = 0.038), and 4 (p = 0.16) were linked to better outcomes. Considering both statistical significance and survival trends, we selected marker genes from clusters 0, 1, and 3—those most strongly associated with prognosis—for subsequent model construction.

### 3.6 From Single Cells to Signatures: Building a Prognostic Model for OV

**Figure 5:**
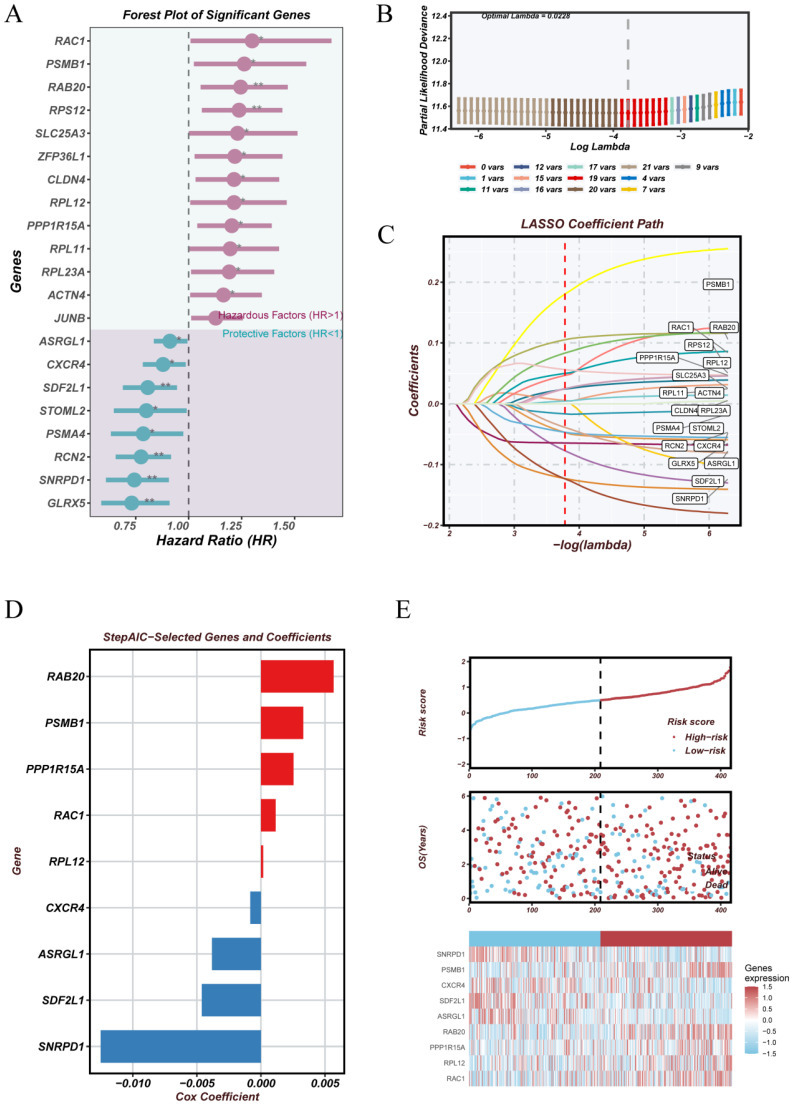
** Construction of a malignant epithelial cell-derived prognostic risk model.** (A)Conducted univariate Cox regression analysis on marker genes identified from clusters 0, 1, and 3. Genes significantly linked to prognosis are displayed, with high-risk factors (HR > 1) highlighted in red and protective factors (HR < 1) in blue. (B) Utilizing ten-fold cross-validation to select tuning parameters in LASSO regression. The optimal lambda value is selected to minimize partial likelihood deviance. (C) LASSO coefficient paths of selected genes across varying penalty parameters, illustrating the shrinkage behavior of model coefficients. (D) Final gene selection using the stepwise AIC algorithm applied to the LASSO results. Bar plot displays the Cox regression coefficients of the retained genes. (E) Risk score distribution and patient stratification. The top section displays risk scores ranked from low to high, the middle section illustrates the distribution of survival status, and the bottom section presents a heatmap of model gene expression profiles across high- and low-risk groups.

To develop a prognostic signature based on malignant epithelial subpopulations, we first identified cluster-specific marker genes from clusters 0, 1, and 3—each showing significant correlation with patient survival in Supplement Figure 1. Figure 5A illustrates the use of univariate Cox regression to assess the prognostic significance of these genes, highlighting risk factors in red and protective factors in blue. Subsequently, LASSO regression was applied to reduce feature redundancy, with ten-fold cross-validation determining the optimal penalty parameter. We selected the minimum value of the tuning parameter (lambda.min = 0.0228) to construct the model (Figure 5B-C).

The resulting multivariate Cox regression model incorporated nine genes with the following formula:

Risk Score = (-0.0038 × ASRGL1) + (-0.0008 × CXCR4) + (0.0025 × PPP1R15A) + (0.0033 × PSMB1) + (0.0057 × RAB20) + (0.0011 × RAC1) + (0.0002 × RPL12) + (-0.0046 × SDF2L1) + (-0.0125 × SNRPD1)

The model coefficients are displayed in Figure 5D, highlighting the relative contribution of each gene. Positive coefficients (e.g., RAB20 and PSMB1) indicate risk-enhancing effects, whereas negative coefficients (e.g., SNRPD1 and SDF2L1) suggest protective roles. Figure 5E displays a heatmap of model gene expression patterns in patients, categorized into high- and low-risk groups according to the median risk score.

To evaluate the stability and generalizability of the constructed prognostic model across different OV cohorts, we conducted validation analyses in four independent datasets: TCGA-OV, GSE13876, GSE140082, and GSE26712. Kaplan-Meier survival analysis in the TCGA-OV cohort showed that patients in the high-risk group had significantly shorter overall survival than those in the low-risk group (p < 0.0001). The AUC values at 1, 3, and 5 years were 0.628, 0.683, and 0.707, respectively, indicating moderate but consistent predictive performance. In the GSE13876 cohort, the model also showed significant prognostic stratification (p < 0.0001), with AUC values of 0.629, 0.677, and 0.686 at 1, 3, and 5 years, respectively. In the GSE140082 cohort, despite the relatively shorter follow-up time, a significant survival difference was observed (p = 0.0068), with AUC values of 0.670 and 0.676 at 1 and 3 years. In the GSE26712 cohort, the model achieved statistically significant survival separation (p = 0.02), with AUC values of 0.577, 0.639, and 0.685. These results support the reproducibility of the risk score across independent datasets, while indicating moderate rather than strong discriminative ability.

### 3.7 Gene Set Enrichment Analysis Highlights Molecular Distinctions Between Risk Groups

**Figure 7:**
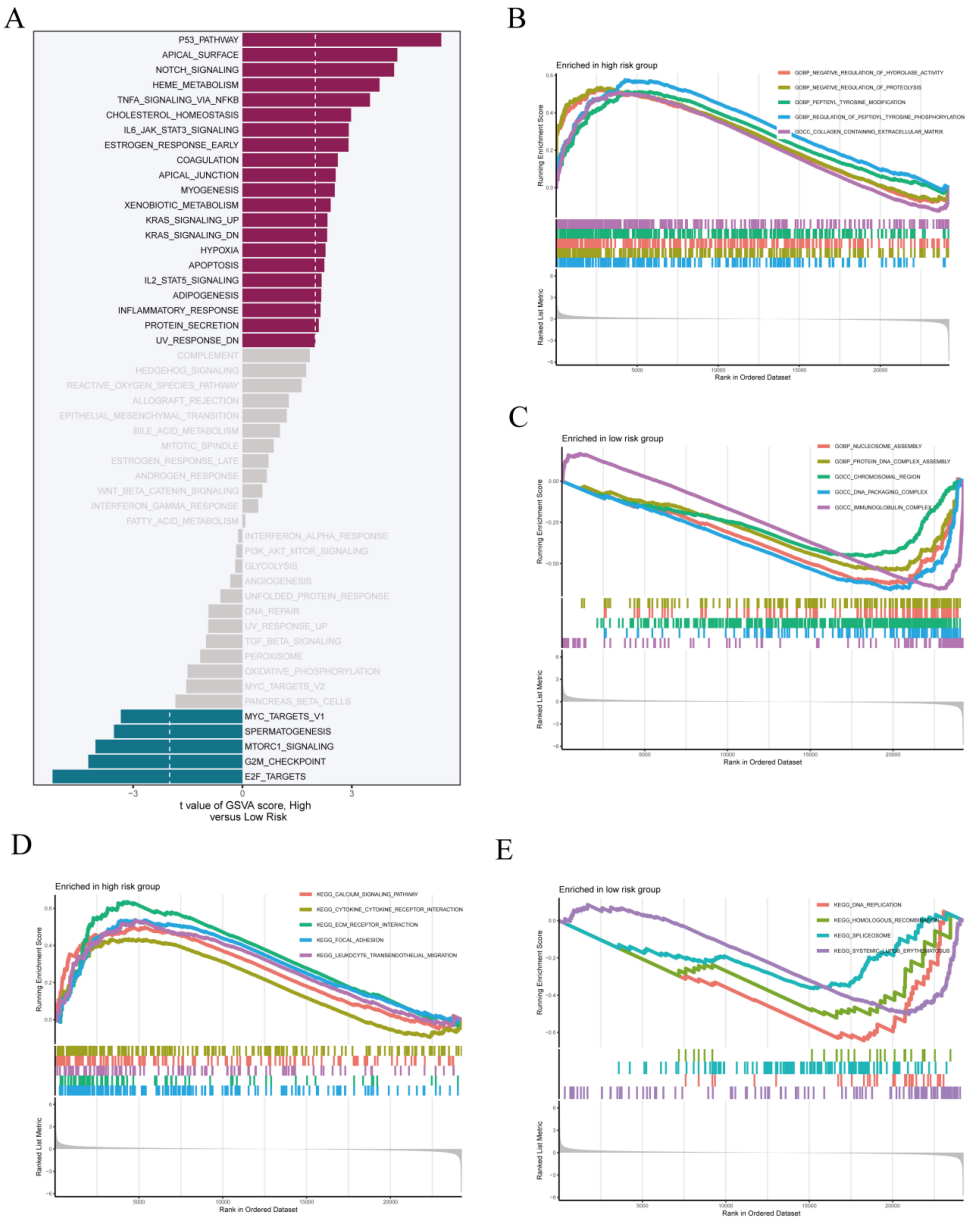
** Functional enrichment analysis comparing high-risk and low-risk groups.** (A) Gene Set Variation Analysis (GSVA) was performed to assess pathway activation differences between high- and low-risk groups, highlighting positively enriched pathways in red and negatively enriched ones in blue. Gene Ontology (GO) enrichment analysis identified key biological processes that were upregulated in both the (B) high-risk group and the (C)low-risk group. KEGG pathway analysis highlighted the significantly enriched pathways in both the high-risk (D) and low-risk (E) groups.

Building upon the previously established prognostic model, we next investigated the functional differences between risk subgroups using GSVA and enrichment analyses. GSVA results revealed increased activity in pathways linked to malignant progression, such as the P53 signaling pathway, apical surface organization, and Notch signaling, in the high-risk group. The low-risk group showed an enrichment of pathways associated with immune homeostasis and metabolic regulation (Figure 7A), indicating distinct functional states between the groups. Gene Ontology enrichment analysis indicated that the high-risk group was mainly linked to the negative regulation of hydrolase activity and proteolysis (Figure 7B). The low-risk group demonstrated notable enrichment in nucleosome assembly and protein-DNA complex assembly processes, which are integral to chromatin remodeling and maintaining nuclear structure (Figure 7C).

At the KEGG pathway level, the high-risk group showed increased activity in immune and signaling pathways, such as the calcium signaling pathway and cytokine-cytokine receptor interaction (Figure 7D). The low-risk group showed enrichment in DNA replication and homologous recombination pathways, indicating improved genome maintenance capacity (Figure 7E). In summary, the two risk subgroups exhibit substantial divergence in their molecular and functional profiles, reinforcing the biological interpretability and robustness of the constructed risk stratification system.

### 3.8 Integrated Evaluation of Risk Model and Tumor Mutation Burden

**Figure 8:**
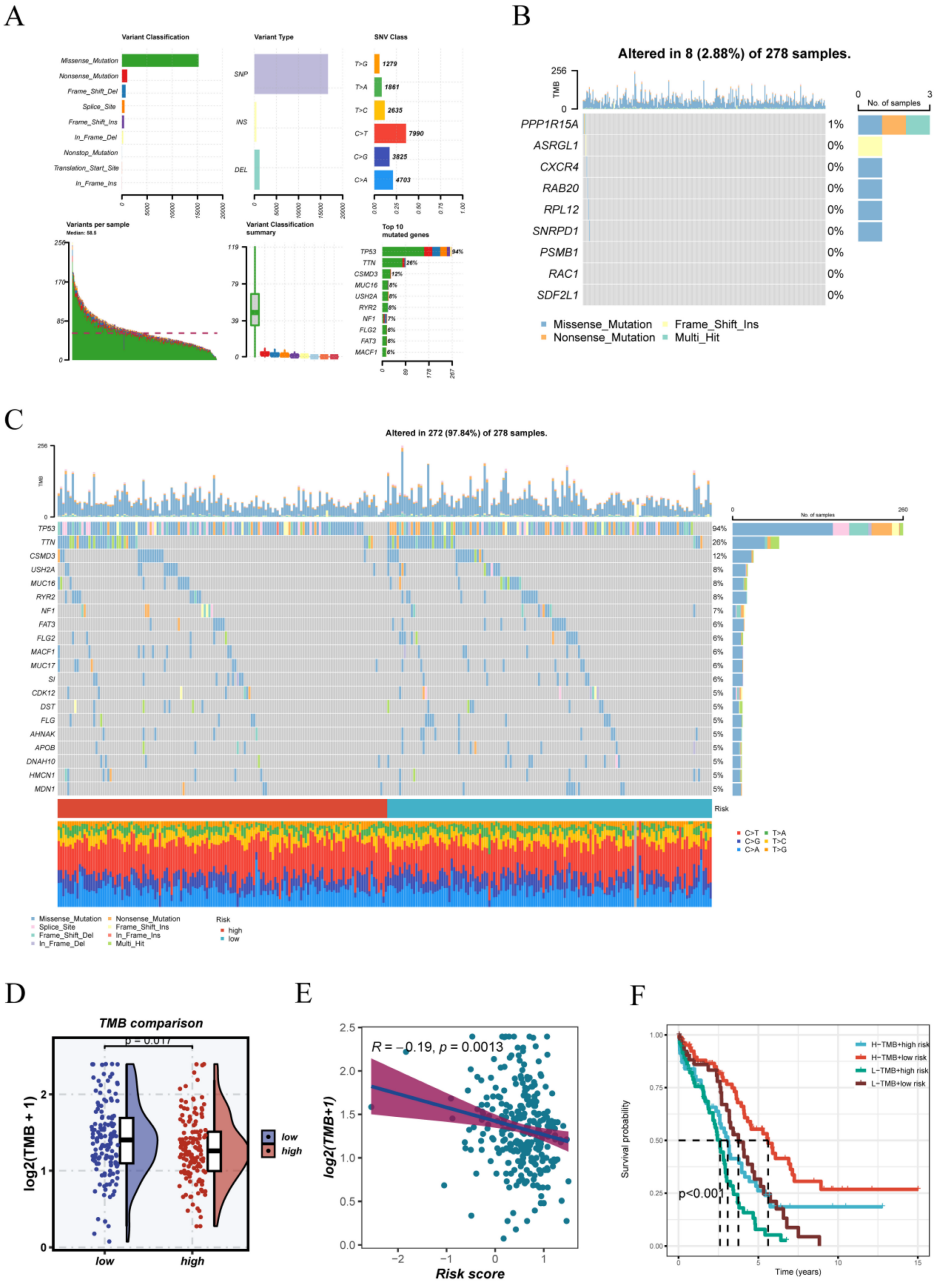
** Somatic mutation and TMB analysis in TCGA-OV cohort.** (A) Summary of somatic mutation profiles in TCGA-OV, including mutation classification, variant types, SNV classes, and top mutated genes. (B) Mutation status of the nine genes included in the prognostic model, with overall mutation frequencies. (C) Oncoplot illustrating the mutation landscape stratified by risk groups; mutation types and clinical annotations are shown below. (D)Tumor mutation burden (TMB) was compared between high- and low-risk groups, revealing that the high-risk group had lower TMB levels. (E) Correlation analysis revealed a significant negative relationship between risk score and TMB. (F) Combined survival analysis of risk score and TMB level.

We investigated the mutational characteristics associated with the risk score in the TCGA-OV cohort (Figure 8A). Among the variant classifications, missense mutations were the most prevalent. Single nucleotide polymorphisms (SNPs) accounted for the majority of variant types, with C>T being the most common single nucleotide variant (SNV) class. The genes TP53, TTN, MUC16, and CSMD3 were among the most commonly mutated, with TP53 exhibiting the highest mutation frequency. The nine genes incorporated into the prognostic model displayed relatively low mutation frequencies, with the highest alteration rate not exceeding 2.88% (Figure 8B).

The analysis of somatic mutation profiles showed distinct patterns between high- and low-risk subgroups, with TP53 mutations being prominent in both (Figure 8C). The analysis revealed that TMB levels were significantly lower in the high-risk group than in the low-risk group (Figure 8D). A notable inverse relationship was identified between TMB and the risk score (R = -0.19, p = 0.0013; Figure 8E). Notably, survival analysis integrating both TMB and risk stratification demonstrated that patients with low risk and high TMB had the most favorable outcomes, whereas those with high risk and low TMB had the worst prognosis (Figure 8F). These findings highlight the potential of integrating TMB with the risk model to enhance prognostic accuracy.

### 3.9 Elevated Risk Scores Coupled with Enhanced Angiogenesis and EMT: Evidence from 33 Cancers

**Figure 9:**
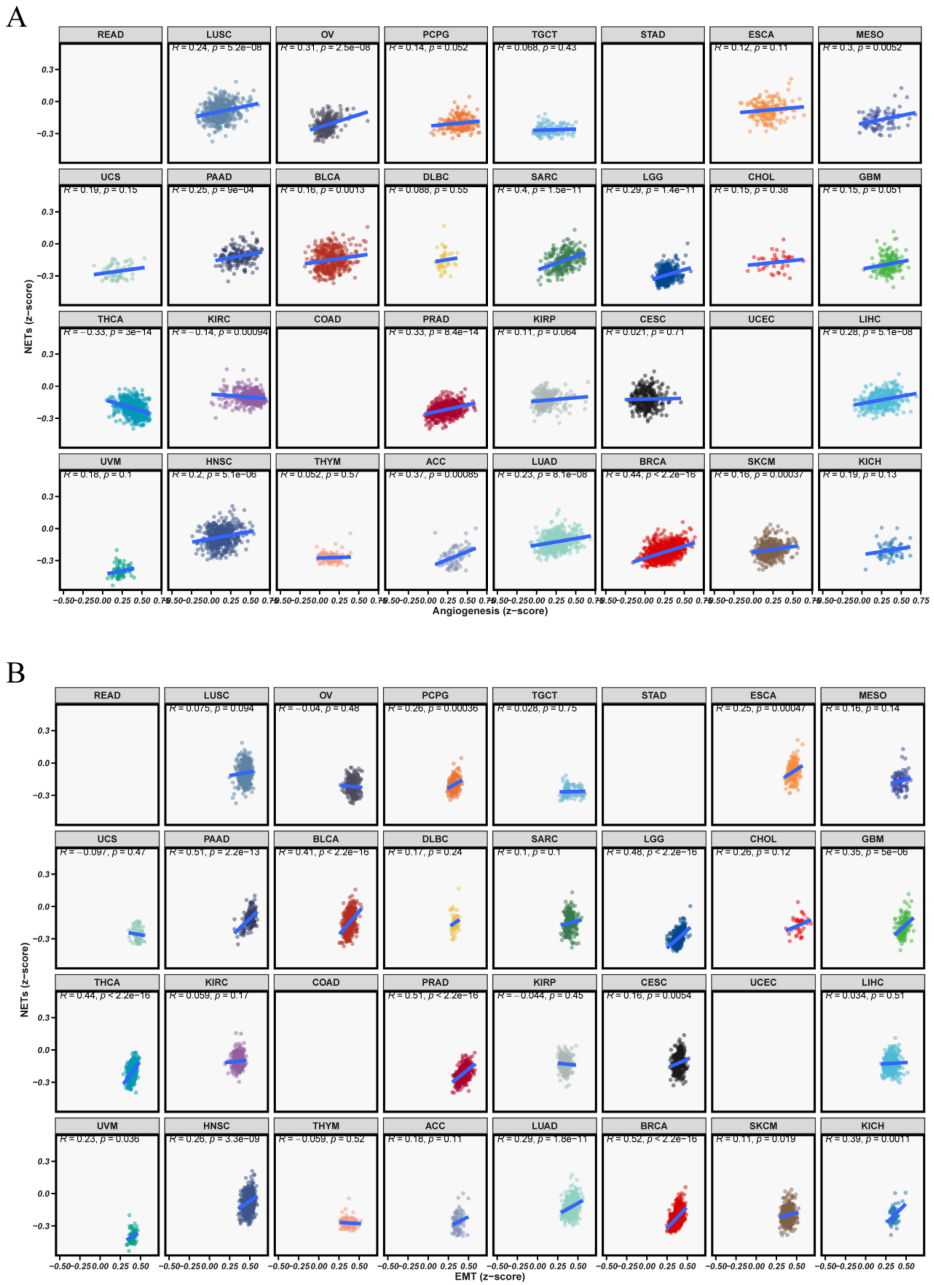
** Association between risk score and tumor phenotype-related pathways in pan-cancer.** (A)Scatter plots illustrating the relationship between risk scores and angiogenesis pathway activity across TCGA pan-cancer cohorts. (B) Scatter plots depicting the correlation between risk score and epithelial-mesenchymal transition (EMT) signature. Each dot represents an individual tumor sample, color-coded by cancer type. Each subplot displays Pearson correlation coefficients and corresponding p-values.

We conducted a pan-cancer correlation analysis to explore the biological significance of the model-derived risk score in relation to hallmark tumor phenotypes, focusing on angiogenesis and epithelial-mesenchymal transition (EMT). Figure 9A illustrates a positive correlation between the risk score and angiogenesis activity in various cancer types, such as OV, LUAD, BRCA, and PAAD. This indicates that patients in the high-risk group might have increased neovascularization potential, which could facilitate tumor progression and metastasis. Figure 9B also shows a strong positive correlation between the risk score and EMT pathway activation in various cancers, including OV, PRAD, KIRC, and LUAD. The results suggest that individuals at high risk are more prone to exhibit aggressive phenotypes with enhanced metastatic potential. Overall, the results highlight that the identified risk signature is not only prognostically relevant but also reflects key malignant characteristics of tumor biology.

### 3.10 Integrating Immune Signatures and Drug Sensitivity Prediction to Assess Clinical Utility

**Figure 10:**
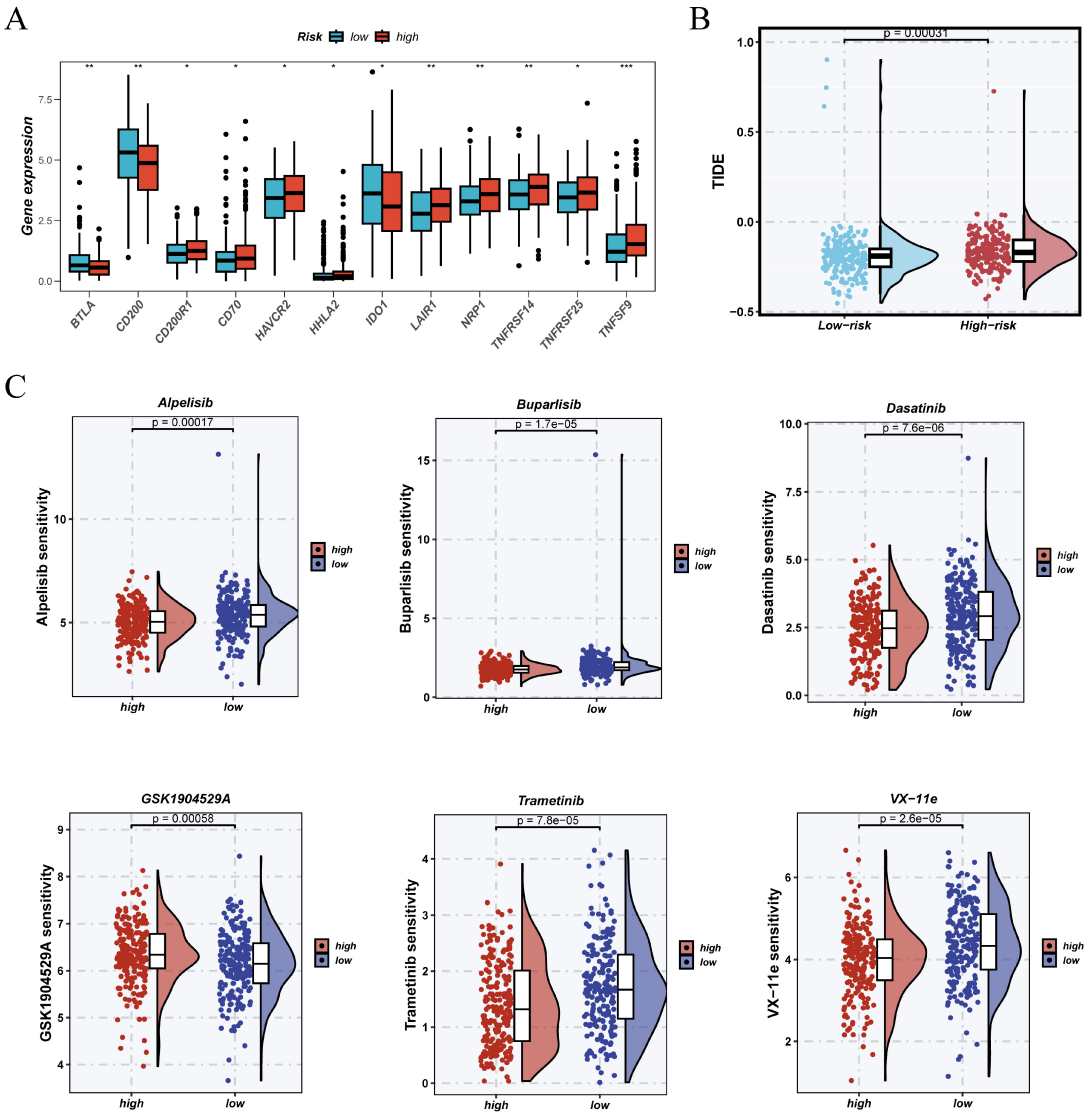
** Potential of the risk score for predicting immunotherapy response and drug sensitivity.** (A)Differential expression of immune checkpoint-related genes was observed between high- and low-risk groups. (B)TIDE analysis revealed that the high-risk group had a greater potential for immune evasion, indicating a possibly poorer response to immunotherapy. (C)The OncoPredict algorithm identified notable variations in drug sensitivity between the two groups.

Expanding on established risk stratification, we examined variations in immunotherapeutic response and drug sensitivity between high- and low-risk groups using immune checkpoint expression analysis, TIDE prediction, and chemotherapeutic sensitivity profiling. Figure 10A illustrates that immune checkpoint-related genes, including HAVCR2, LAIR1, TNFRSF14, TNFRSF25, and TNFSF9, are significantly upregulated in the high-risk group, suggesting an immunosuppressive or exhausted immune microenvironment.

TIDE analysis (Figure 10B) indicated that high-risk patients generally had elevated TIDE scores, implying a greater chance of immune evasion and diminished response to immune checkpoint blockade therapies. The OncoPredict algorithm's drug sensitivity profiling (Figure 10C) revealed that high-risk patients had increased IC50 values for several agents, such as Alpelisib, Buparlisib, Dasatinib, GSK1904529A, Trametinib, and VX-11e, suggesting reduced predicted sensitivity to these treatments. These findings suggest that high-risk individuals may exhibit an immune-suppressive phenotype and diminished therapeutic response, underscoring the necessity for alternative or combination treatment strategies.

### 3.11 Expression Profile and Functional Validation of Tumor-Promoting Gene PSMB1

**Figure 11:**
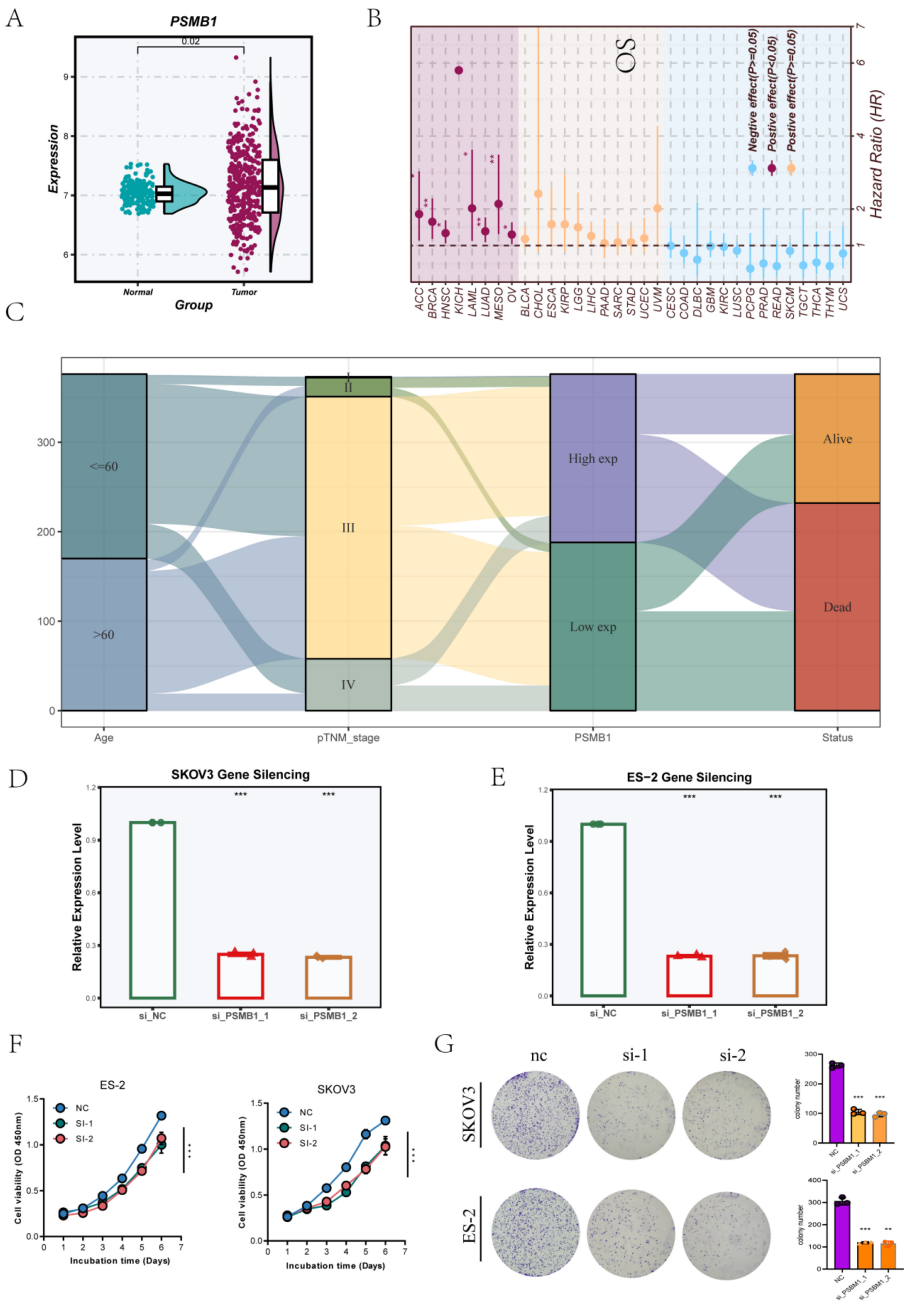
** Expression Profile and Functional Validation of Tumor-Promoting Gene PSMB1.** (A)Analysis of GTEx and TCGA datasets showed significant overexpression of PSMB1 in tumor tissues. (B)Pan-cancer survival analysis revealed that elevated PSMB1 expression correlates with poor prognosis in various cancer types. (C)The Sankey diagram based on TCGA data depicted the associations among PSMB1 expression, age, TNM stage, and survival status. (D-E)siRNA knockdown models of PSMB1 were effectively created in SKOV3 and ES-2 OV cell lines, with gene silencing efficiency verified by qRT-PCR. (F) CCK-8 assays demonstrated that PSMB1 knockdown suppressed cell proliferation. (G) Colony formation assays further validated the tumor-promoting role of PSMB1.

During model development, PSMB1 emerged as a key gene of interest due to its consistently high hazard ratio in univariate Cox regression analysis and its relatively large coefficient in the final risk model, indicating its substantial contribution to risk stratification. Therefore, we selected PSMB1 for further experimental investigation. Analysis of GTEx and TCGA datasets showed significantly higher PSMB1 expression in tumor tissues than in normal controls (Figure 11A). Pan-cancer survival analysis further demonstrated that high PSMB1 expression was associated with worse prognosis across multiple cancer types, underscoring its potential as a prognostic biomarker (Figure 11B). Figure 11C presents a Sankey diagram using TCGA data, showing the relationships between PSMB1 expression and clinical variables, including age, TNM stage, and survival status.

To explore its functional role, siRNA-mediated knockdown of PSMB1 was established in SKOV3 and ES-2 OV cell lines, with qRT-PCR confirming effective gene silencing (Figure 11D-E). CCK-8 assays demonstrated that PSMB1 silencing markedly inhibited cell proliferation (Figure 11F). Colony formation assays demonstrated a significant decrease in tumorigenic potential following PSMB1 knockdown (Figure 11G), indicating that PSMB1 functions as a tumor-promoting gene in OV cells.

## 4. Discussion

OV is among the deadliest female reproductive system malignancies, primarily due to its subtle onset and the absence of effective early screening methods 22. As a result, most patients are diagnosed at advanced stages 23. Despite improvements in surgical techniques, chemotherapy, and the introduction of targeted and immunotherapeutic options, overall survival rates remain unsatisfactory, with five-year survival hovering below 30% 24. In this context, the development of reliable molecular biomarkers and risk stratification tools is urgently needed to support personalized treatment approaches.

This study developed a single-cell transcriptomic atlas of epithelial cells in OV, identifying subpopulations with different malignant potentials. We then extracted subcluster-specific marker genes and applied machine learning algorithms to develop a biologically grounded, nine-gene risk score system. The model showed moderate but consistent prognostic performance across independent cohorts and was associated with multi-omics features, including mutation burden, immune microenvironment traits, and therapeutic responsiveness, indicating its potential translational relevance. This observation is consistent with recent evidence that malignant cell clusters identified by single-cell or multi-omics analysis may provide clinically relevant information for prognostic interpretation and therapeutic exploration 25.

Among the genes incorporated in the model, ASRGL1 has been implicated in cellular metabolism, and although upregulated in some solid tumors, its expression in this study was associated with favorable prognosis, potentially reflecting context-dependent roles in specific epithelial subsets 26, 27. CXCR4, a chemokine receptor broadly involved in metastasis and immune evasion, exhibited a negative coefficient here, suggesting tissue-specific heterogeneity in its prognostic impact 28. PPP1R15A, known for its role in stress response and translational control, was associated with poor outcomes, possibly due to its protective effects on tumor cells under adverse conditions 28-30. PSMB1, the focus of our functional validation, encodes a proteasome subunit involved in cell cycle progression and apoptosis inhibition 31. Its involvement in tumor progression across various cancers has been suggested, and we validated its oncogenic role in OV using qRT-PCR, CCK-8, and colony formation assays.

RAB20, a member of the Ras GTPase family, is linked to vesicular transport and cell migration, and may promote invasive behavior in tumor cells 32, 33. RAC1, a well-characterized Rho GTPase, drives cytoskeletal remodeling and has been validated as a pro-tumorigenic factor in various cancers 34. RPL12 encodes a ribosomal protein and, although underexplored, may support tumor cell proliferation through enhanced protein synthesis, as suggested by its positive association with poor prognosis 35. SDF2L1, an endoplasmic reticulum-associated protein involved in stress response and protein folding, emerged as a protective factor, possibly due to its role in maintaining cellular homeostasis 36, 37. SNRPD1, a component of the spliceosomal complex, has been associated with tumor cell proliferation and unfavorable outcomes in several cancers 38, 39. However, in the present OV-derived risk model, SNRPD1 showed a negative coefficient, suggesting that its prognostic effect may be context-dependent and may reflect the specific transcriptional state captured by this malignant epithelial cell-derived signature.

From a translational perspective, our risk score model offers several advantages. It is biologically interpretable due to its derivation from well-defined malignant epithelial subpopulations. It demonstrated consistent predictive performance in both TCGA and multiple external validation cohorts. Furthermore, its associations with clinical features, immune infiltration, mutation profiles, and drug sensitivity highlight its potential utility in guiding personalized therapy.

Nonetheless, this study has limitations. Although the model was validated in publicly available cohorts, prospective clinical validation in larger and more diverse populations is necessary to confirm its generalizability. Moreover, the accuracy of cell subpopulation identification and batch correction may influence downstream gene selection and model stability. Although PSMB1 knockdown was shown to inhibit OV cell proliferation and colony formation, the current experimental validation was limited to *in vitro* growth-related assays. Further studies incorporating migration, invasion, apoptosis, rescue experiments, protein-level validation, and *in vivo* tumor models are warranted to more comprehensively define the functional role and therapeutic relevance of PSMB1 in OV. Future studies integrating spatial transcriptomics, proteomics, and metabolomics could provide deeper insights into the biology of key subpopulations and uncover novel therapeutic targets. In addition, TIDE-based immunotherapy prediction and OncoPredict-based drug sensitivity analysis should be interpreted as exploratory computational estimates rather than direct evidence of clinical treatment response. These findings require further validation in ovarian cancer cohorts with documented immunotherapy outcomes, pharmacological response data, and experimental models.

In summary, this study developed a robust epithelial cell-derived prognostic model for OV by integrating single-cell and bulk transcriptomic data. The model provides reproducible risk stratification and also sheds light on the biological mechanisms and therapeutic implications of tumor heterogeneity, offering a potentially useful framework for precision oncology in OV.

## Supplementary Material

Supplementary figures and tables.

## Figures and Tables

**Figure 6:**
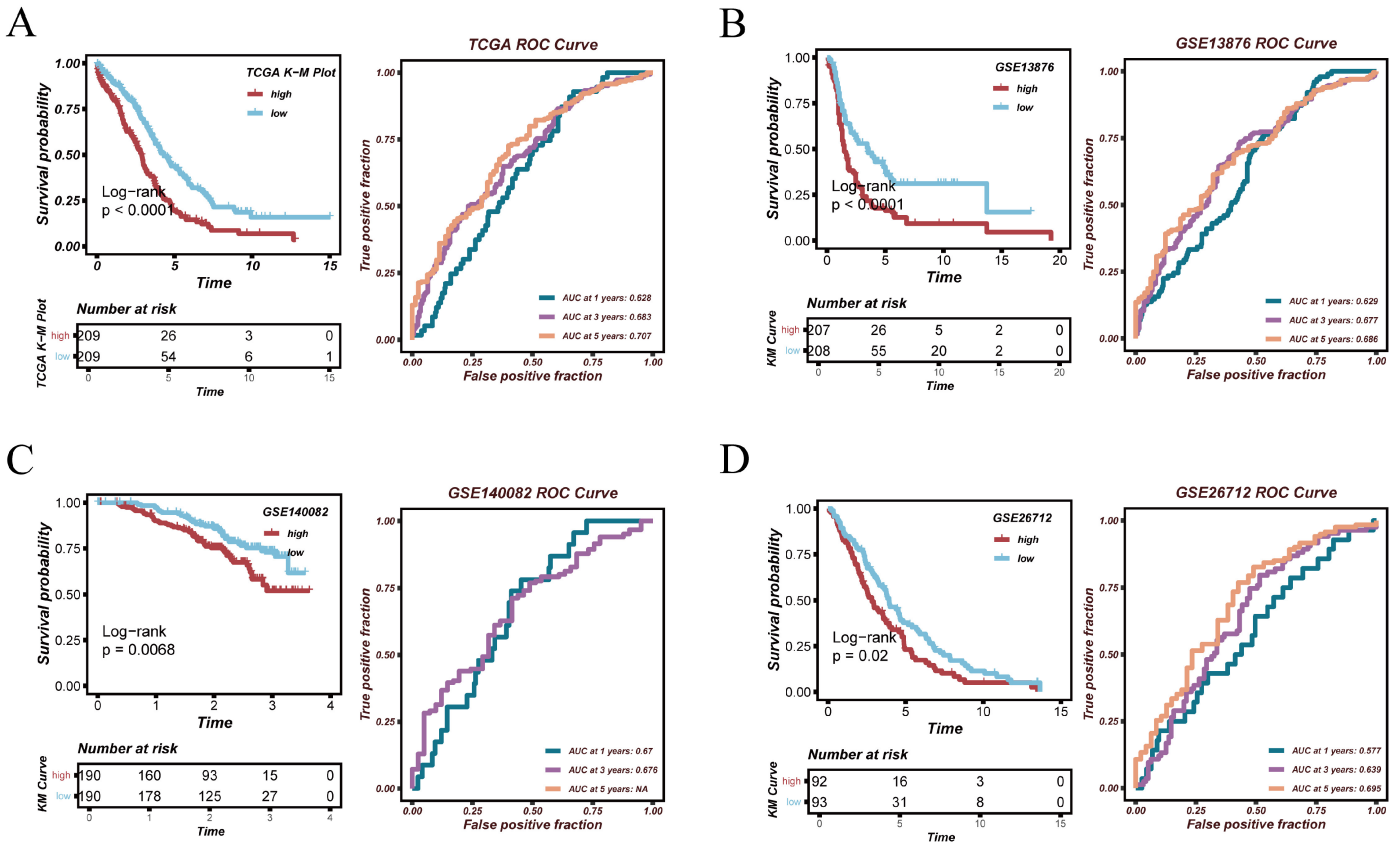
** Validation of Prognostic Performance Across Multiple Cohorts.** (A) Kaplan-Meier survival analysis and time-dependent ROC evaluation were conducted on the TCGA-OV cohort. (B) Survival analysis and ROC curves in the GSE13876 validation cohort. (C)Prognostic validation in the GSE140082 cohort was conducted using Kaplan-Meier analysis and receiver operating characteristic (ROC) evaluation. (D) External validation in the GSE26712 dataset, showing survival difference and predictive performance. Patients were categorized into high- and low-risk groups according to the median risk score. AUC values for 1-, 3-, and 5-year survival were calculated to assess the predictive accuracy of the model.

## Data Availability

All data used in this study are publicly available. The single-cell RNA sequencing datasets were obtained from the Gene Expression Omnibus (GEO) database under accession numbers GSE154600 and GSE184880. Bulk RNA-seq datasets used for external validation were also retrieved from GEO, including GSE13876, GSE140082, and GSE26712. The TCGA-OV cohort was downloaded from The Cancer Genome Atlas (TCGA) via the UCSC Xena platform (https://xena.ucsc.edu/). Somatic mutation data (MAF files) were obtained from the Genomic Data Commons (GDC) portal (https://portal.gdc.cancer.gov/). TIDE (Tumor Immune Dysfunction and Exclusion) scores were obtained from the TIDE web server (http://tide.dfci.harvard.edu/) for immunotherapy response prediction. All relevant data are publicly accessible from the above-mentioned repositories. Further information can be obtained from the corresponding author upon reasonable request.
