# Supplementary material for: Single-Cell-Derived Malignant Epithelial Programs Define Prognostic Risk and Therapeutic Vulnerability in Ovarian Cancer
Source: J Cancer. 2026 Jul 3;17(7):1274–94. doi: 10.7150/jca.135548 (PMC13410796; doi:10.7150/jca.135548)
Supplement: Supplementary file 1 — Supplementary figures and tables. [file jcav17p1274s1.pdf]

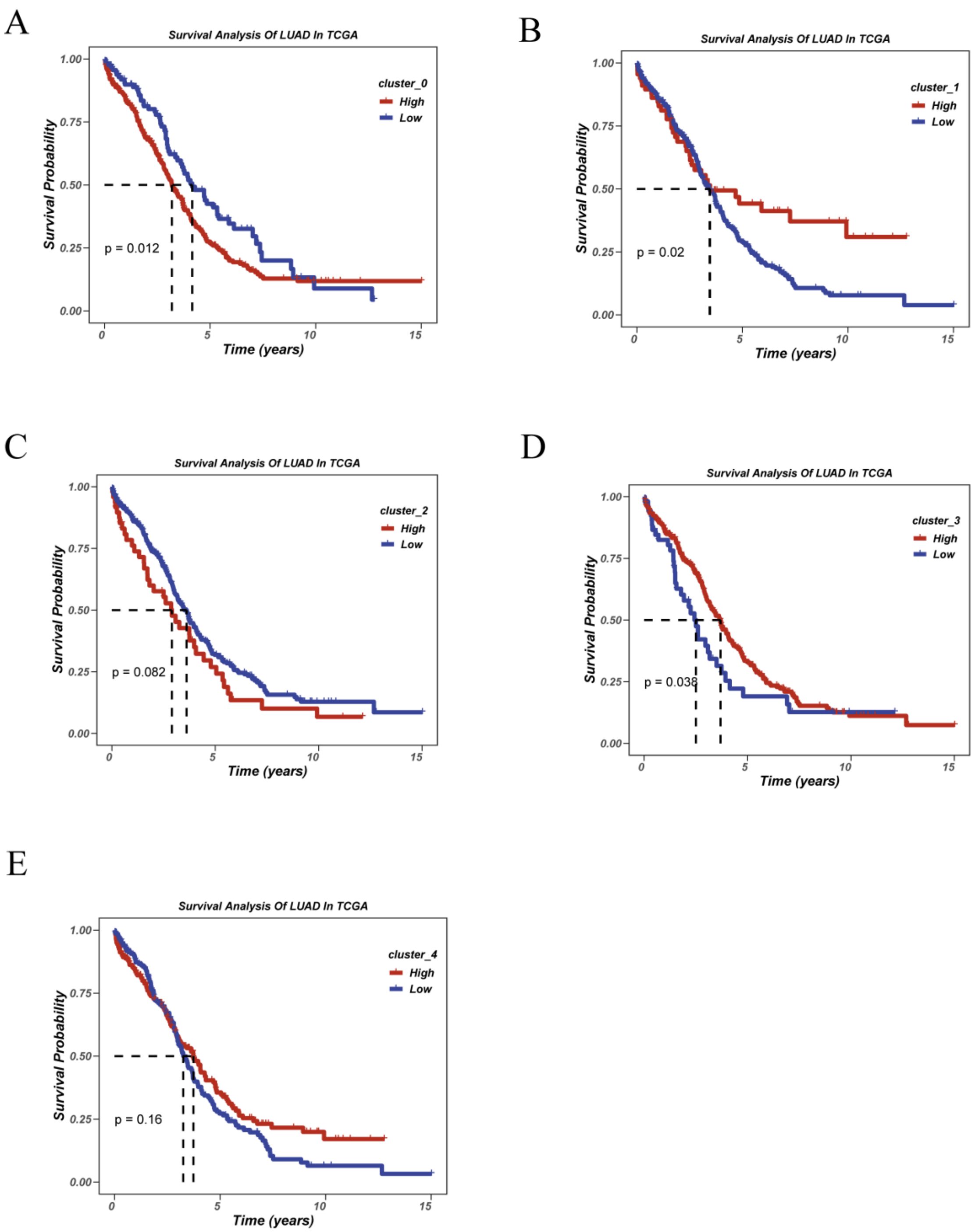

Supplementary Figure 1. Prognostic significance of malignant epithelial subpopulations (clusters 0–4) in the TCGA cohort. Kaplan–Meier survival curves (A–E) depict overall survival variations between high- and low-score groups across clusters 0 to 4 in the TCGA-OV cohort. Patients were stratified based on the median enrichment score of each cluster-specific gene set. Statistical significance was assessed using the log-rank test.

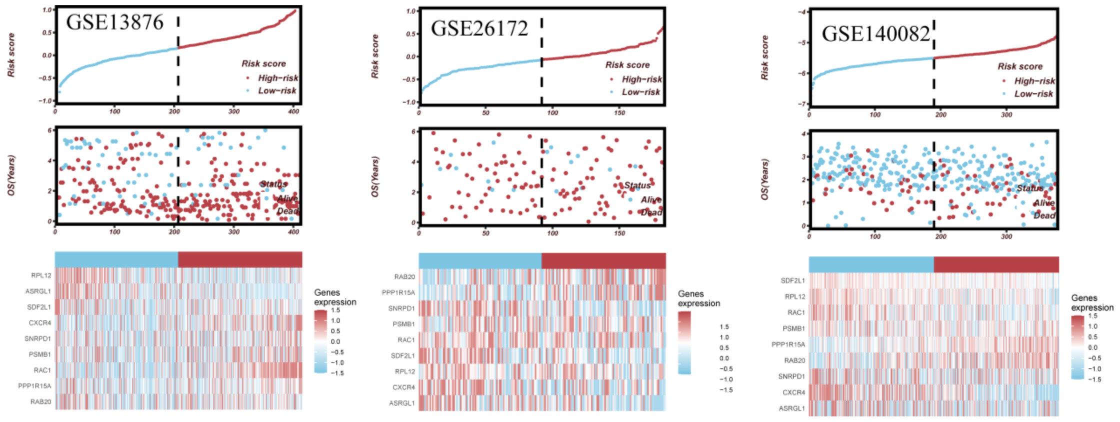

Supplement Figure 2. Risk distribution plots, survival status scatter plots, and gene expression heatmaps were generated for high- and low-risk groups in the validation cohorts GSE13876, GSE26172, and GSE140082.

Supplementary Table 1. Quality control thresholds for single-cell RNA sequencing.

Filtering Parameter	Threshold Value
nFeature_lower	500
nFeature_upper	10000
nCount_lower	1000
nCount_upper	100000
pMT_lower	0
pMT_upper	40
pHB_lower	0
pHB_upper	5

Supplementary Table 1. Thresholds applied for single-cell RNA-seq quality control
